# GSDMD is dispensable for hematopoietic homeostasis and hematopoietic stem cell function

**DOI:** 10.1093/lifemedi/lnac025

**Published:** 2022-08-01

**Authors:** Tingting Cong, Yuqian Wang, Jianwei Wang

**Affiliations:** School of Pharmaceutical Sciences, Tsinghua University, Beijing 100084, China; School of Pharmaceutical Sciences, Tsinghua University, Beijing 100084, China; School of Pharmaceutical Sciences, Tsinghua University, Beijing 100084, China

**Dear Editor,**

Hematopoietic stem cell (HSC) builds up the hematopoietic system by generating all blood cells including myeloid lineage (monocytes, macrophages, basophils, neutrophils, eosinophils, erythrocytes, megakaryocytes, platelets, and dendritic cells) and the lymphoid lineage (T cells, B cells, and NK cells). The homeostasis of the hematopoietic system is strictly regulated by various mechanisms [1]. Growing evidence shows that programmed cell death signaling, including apoptosis, necroptosis, and pyroptosis, play a vital role in modulating HSC function [2, 3]. Pyroptosis is executed by gasdermin family proteins, including GSDMA, GSDMB, GSDMC, GSDMD, and GSDME [4].

GSDMD is widely expressed in different tissues and cells of humans and mammals [5]. Human GSDMD is highly expressed in immune cells, while murine GSDMD is mainly distributed in immune cells and intestinal epithelial cells [6]. The previous study revealed that the N-terminal fragment of GSDMD rapidly bind membrane lipids to exhibit membrane-disrupting cytotoxicity in mammalian cells [4, 7]. Several studies reported that Caspase-4/5/11 in the cytoplasm can be used as a sensor to directly bind to the surface LPS of gram-negative bacteria, and mediate the nonclassical pathway of cell pyrolysis and GSDMD is one of the key executive molecules mediating pyrolysis [8]. GSDMD-mediated pyrolysis eliminates pathogens, promote damage repair, and maintain homeostasis [5]. On the other hand, uncontrolled pyrolysis can cause tissue pathological damage, and various diseases, such as sepsis, inflammatory bowel disease, atherosclerosis, and so on [9]. In addition, more studies revealed that GSDME, another member of the gasdermin family, is cleaved by Caspase-3 to execute pyroptosis [10].

Our previous studies have reported that GSDME maintains HSCs by balancing pyroptosis and apoptosis [2, 3]. While, whether GSDMD participates in modulating hematopoietic homeostasis or HSC function remains unknown. In this study, we observed no significant difference of hematopoietic system at steady state and upon LPS stimulation between *Gsdmd*^*−/−*^ and control mice. Moreover, the reconstitution capacity and differentiation potential of *Gsdmd*^*−/−*^ HSCs is comparable to WT HSCs.

By exploring the database BioGPS (http://biogps.org/#goto=welcome), we observed that GSDMD is highly expressed in hematopoietic and intestinal system (Fig. 1A), which suggests that GSDMD might play a functional role within it. To test this hypothesis, we imported Gsdmd knockout mice (hereafter named *Gsdmd*^*−/−*^) and performed western blot to examine the deletion efficiency of GSDMD, and the result revealed that GSDMD is deleted in hematopoietic stem and progenitor cells (Fig. 1B).

**Figure 1.**
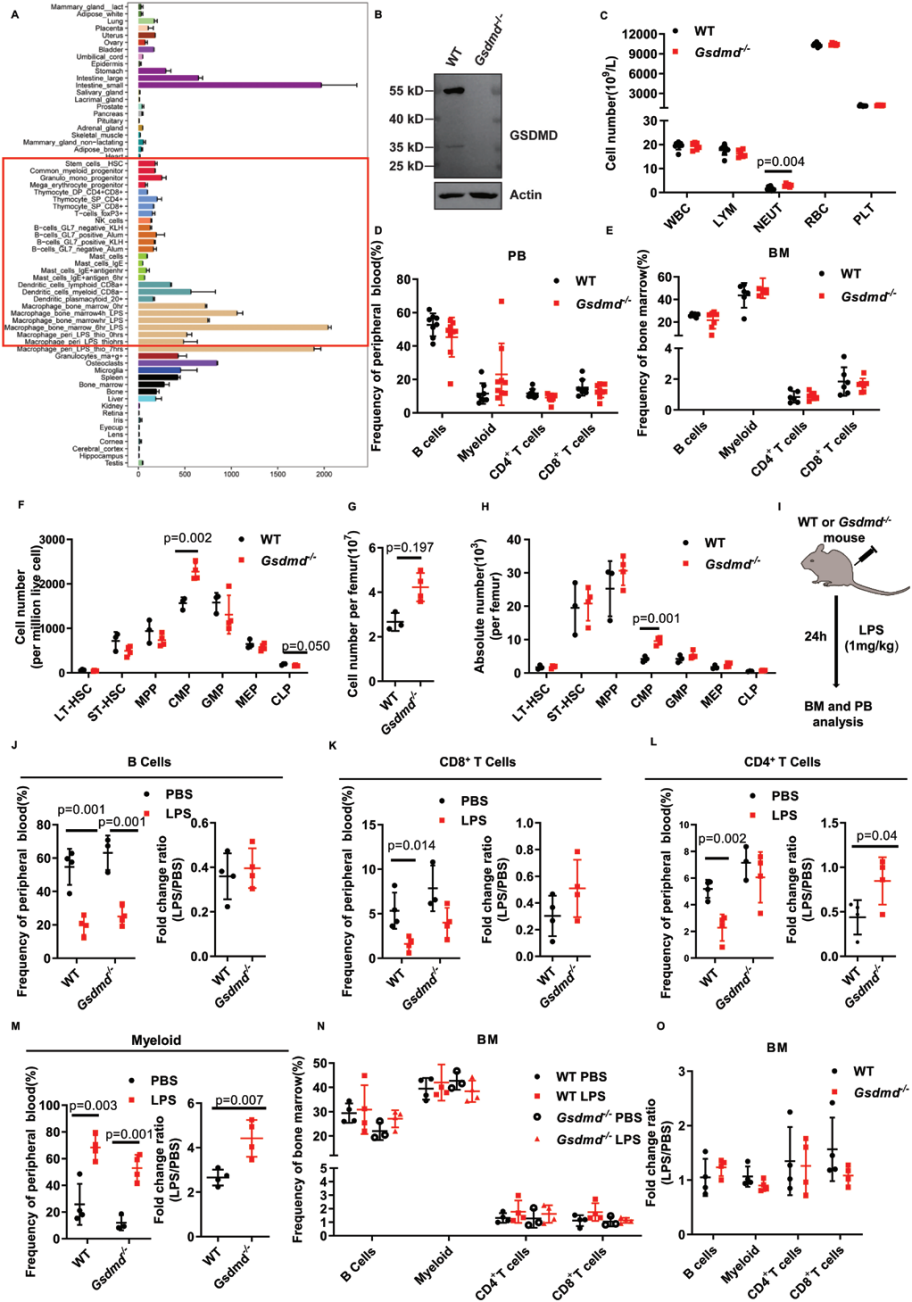
GSDMD is dispensable for hematopoietic homeostasis and LPS-stimulated hematopoietic homeostasis. (A) The expression of GSDMD in different tissues. (B) The western blot displays the expression of GSDMD in hematopoietic stem and progenitor (c-Kit^+^) cells’ lysates of wild-type (WT) mice and *Gsdmd*^−/−^ (KO) mice. (C) The general count analysis shows the number of WBC, LYM, NEUT, RBC, and PLT in WT mice and *Gsdmd*^−/−^ mice. (*n* = 6–8 mice per group. Data are shown as mean ± SD.) (D) The scatter plots depict the percentage of B cells (B220^+^), myeloid (Mac-1^+^), CD4^+^ T cells (CD4^+^CD8^−^), and CD8^+^ T cells (CD4^−^CD8^+^) in PB of 2-month-old WT and *Gsdmd*^−/−^ mice. (*n* = 8–9 mice per group. Data are shown as mean ± SD.) (E) The scatter plots depict the percentage of B cells (B220^+^), myeloid (Mac-1^+^), CD4^+^ T cells (CD4^+^CD8^−^), and CD8^+^ T cells (CD4^−^CD8^+^) in BM of 2-month-old WT and *Gsdmd*^−/−^ mice. (*n* = 6–7 mice per group. Data are shown as mean ± SD.) (F) The scatter plots display the frequency of LT-HSC (Lineage^–^Sca-1^+^c-Kit^+^CD135^-^CD34^−^), ST-HSC (Lineage^–^Sca-1^+^c-Kit^+^CD135^−^CD34^+^), MPP (Lineage^–^Sca-1^+^c-Kit^+^CD135^+^CD34^+^), CMPs (Lineage^–^Sca-1^–^c-Kit^+^CD16/32^–^CD34^+^), GMPs (Lineage^–^Sca-1^–^c-Kit^+^CD16/32^+^CD34^+^), MEPs (Lineage^–^Sca-1^–^c-Kit^+^CD16/32^–^CD34^–^), and CLPs (Lineage^–^Sca-1^low^c-Kit^low^ CD135^+^ CD127^+^) in WT and *Gsdmd*^−/−^ mice. (*n* = 3–4 mice per group. Data are shown as mean ± SD.) (G) The scatter plots display the BM cell number per femur of 2-month-old WT and *Gsdmd*^−/−^ mice. (*n* = 3–4 mice per group. Data are shown as mean ± SD.) (H) The scatter plots display the absolute numbers of LT-HSC (Lineage^–^Sca-1^+^c-Kit^+^CD135^−^CD34^−^), ST-HSC (Lineage^–^Sca-1^+^c-Kit^+^CD135^−^CD34^+^), MPP (Lineage^–^Sca-1^+^c-Kit^+^CD135^+^CD34^+^), CMPs (Lineage^–^Sca-1^–^c-Kit^+^CD16/32^–^CD34^+^), GMPs (Lineage^–^Sca-1^–^c-Kit^+^CD16/32^+^CD34^+^), MEPs (Lineage^–^Sca-1^–^c-Kit^+^CD16/32^–^CD34^–^), and CLPs (Lineage^–^Sca-1^low^c-Kit^low^ CD135^+^ CD127^+^) in WT and *Gsdmd*^−/−^ mice. (*n* = 3–4 mice per group. Data are shown as mean ± SD.) (I) WT and *Gsdmd*^*−/−-*^ mice were injected with LPS (1 mg/kg) intraperitoneally, then the mice were analyzed after 24 h. (*n* = 3–4 mice per group, data are shown as mean ± SD.) (J) The scatter plots display the frequency (left histogram) and fold change (right histogram) of B cell in PB. (K) The scatter plots display the frequency (left histogram) and fold change (right histogram) of CD8^+^ T cells in PB. (L) The scatter plots display the frequency (left histogram) and fold change (right histogram) of CD4^+^ T cells in PB. (M) The scatter plots display the frequency (left histogram) and fold change (right histogram) of myeloid cells in PB. (N) The scatter plots display the percentage of B cells (B220^+^), myeloid (Mac-1^+^), CD4^+^ T cells (CD4^+^CD8^−^), and CD8^+^ T cells (CD4^−^CD8^+^) in BM. (*n* = 3–4 mice per group. Data are shown as mean ± SD.) (O) The scatter plots display the relative fold change of B cells (B220^+^), myeloid (Mac-1^+^), CD4^+^ T cells (CD4^+^CD8^−^), and CD8^+^ T cells (CD4^−^CD8^+^) in BM. (*n* =3–4 mice per group. Data are shown as mean ± SD).

We then performed complete blood count assay for *Gsdmd*^*−/−*^ and age-matched control mice. The results revealed no difference of white blood cell (WBC), lymphocyte (LYM), red blood cell (RBC), and platelet (PLT) between *Gsdmd*^*−/−*^ and control mice, while the neutrophil (NEUT) is significantly increased in *Gsdmd*^*−/−*^ mice (Fig. 1C). We then sought to investigate the lineage composition in peripheral blood (PB) and bone marrow (BM) of *Gsdmd* ^*−/−*^ mice, including B cell (B220^+^), myeloid cell (CD11b^+^), CD4^+^ T cell, and CD8^+^ T cell. The results reveal no difference of *Gsdmd* ^*−/−*^ mice compared to WT in PB (Fig. 1D) and BM (Fig. 1E).

We next analyzed hematopoietic stem and progenitor cells, including common myeloid progenitor (CMP), granulocyte-macrophage progenitor (GMP), megakaryocyte-erythroid progenitor (MEP), common lymphoid progenitor (CLP), multipotent progenitor cell (MPP), long-term HSC (LT-HSC), and short-term-HSC (ST-HSC). The result shows that the frequency of LT-HSC, ST-HSC, MPP, GMP, and MEP exhibits no significant difference between *Gsdmd*^*−/−*^ and control mice (Fig. 1F). While, the percentage of CMP is significantly increased and the percentage of CLP is significantly decreased in *Gsdmd*^*−/−*^ mice (Fig. 1F).

We observed the BM cellularity is mildly increased in *Gsdmd*^*−/−*^ mice (Fig. 1G). We then calculated the absolute number of hematopoietic stem and progenitor cells. The results show that there is no difference of LT-HSC, ST-HSC, MPP, GMP, MEP, and CLP between *Gsdmd*^*−/−*^ and control mice (Fig. 1H), while the absolute number of CMP is significantly increased in *Gsdmd*^*−/−*^ mice (Fig. 1H).

A previous study reported that GSDMD functions as the substrate of caspases (caspase-1, murine caspase-11, and human caspase-4/5) to mediate pyroptosis [8]. We then wondered whether *Gsdmd*^*−/−*^ mice exhibit different response in response to LPS stimulation compared to WT mice. To address this hypothesis, we challenged *Gsdmd*^*−/−*^ and WT mice by lipopolysaccharides (LPS) (1 mg/kg). Twenty-four hours later, all mice were sacrificed to analyze the lineage composition in PB and BM (Fig. 1I). The result shows that B cell in PB drops significantly upon LPS stimulation in both *Gsdmd*^*−/−*^ and WT mice (Fig. 1J, left histogram) and there is no difference of fold change between them (Fig. 1J, right histogram). Both CD8^+^ and CD4^+^ T cells drops upon LPS stimulation in both groups (Fig. 1K and 1L, left histogram), and no difference of fold change of CD8^+^ T cells between *Gsdmd*^*−/−*^ and WT mice was observed (Fig. 1K, right histogram), while CD4^+^ T cells of *Gsdmd*^*−/−*^ mice exhibit significantly resistance to LPS-induced reduction (Fig. 1L, right histogram). It is notable that myeloid cells increases upon LPS stimulation in both *Gsdmd*^*−/−*^ and WT mice, while the myeloid cells of *Gsdmd*^*−/−*^ mice exhibits significantly expansion in response to LPS stimulation (Fig. 1M). In addition, the percentage and fold change of the aforementioned lineages exhibits no difference in BM between *Gsdmd*^*−/−*^ and WT mice (Fig. 1N and 1O).

To evaluate the impact of Gsdmd deficiency on the long-term hematopoietic reconstitution capacity of HSCs, we performed competitive transplantation assays. Freshly isolated HSCs from WT or *Gsdmd*^*−/−*^ mice were injected into lethally irradiated recipient mice together with 2.5 × 10^5^ total BM cells as competitor (Fig. 2A). Chimera in PB was evaluated every 4 weeks until the 12th week. We observed that the chimera of *Gsdmd*^*−/−*^-derived cells exhibits no difference compare to control group (Fig. 2C), neither to lineage distribution (Fig. 2B).

**Figure 2.**
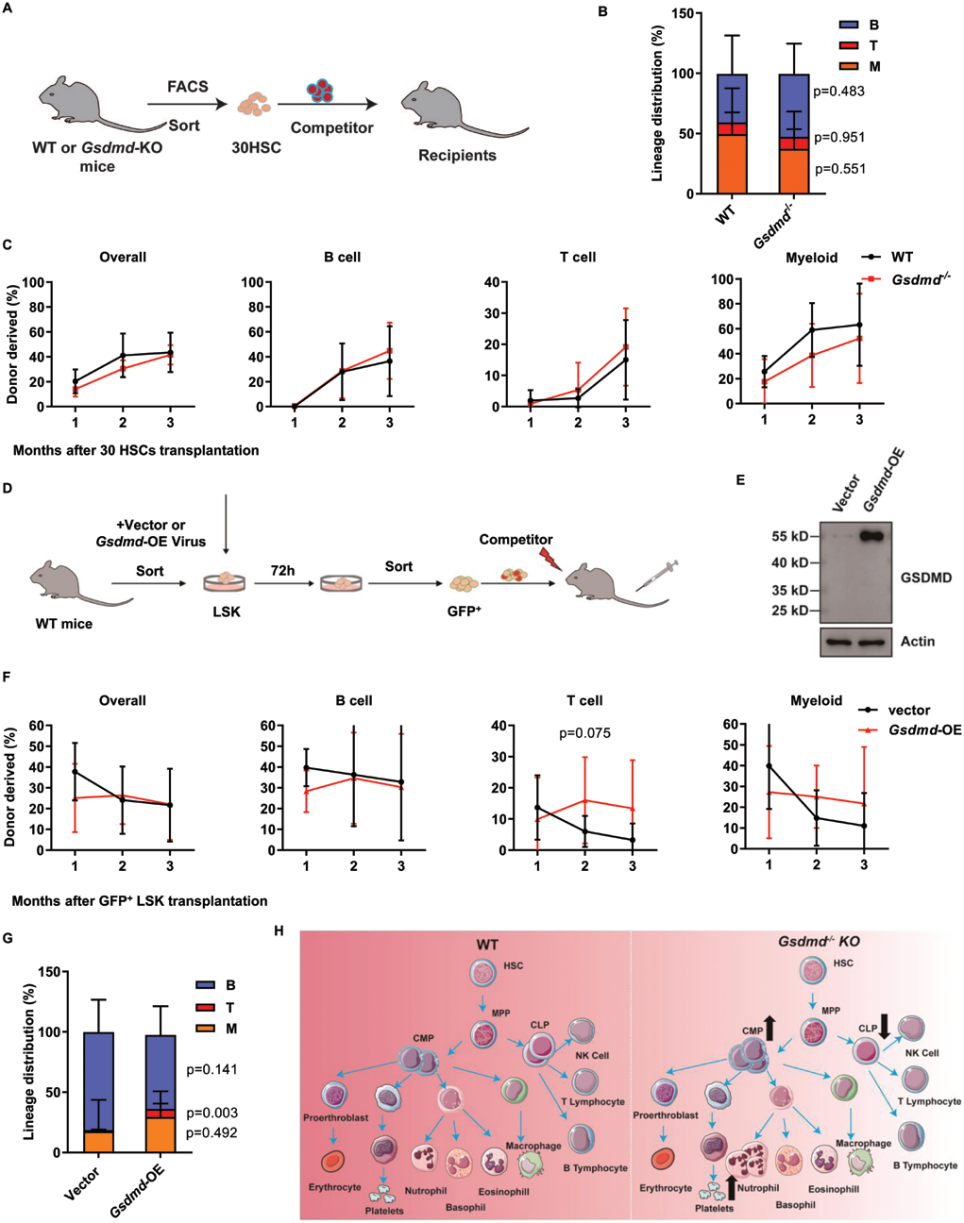
The reconstitution capacity of *Gsdmd*^*−/−*^ and *Gsdmd*-overexpressed HSCs is comparable as WT HSCs. (A) Scheme of transplantation strategy for *Gsdmd*^−/−^ mice. Freshly isolated 30 HSCs from 2 months WT or *Gsdmd*^−/−^ mice were transplanted into lethally irradiated recipients along with 2.5 × 10^5^ competitor cells. Chimera in PB was checked every month. (B) The lineage distribution of indicated donor-derived PB cells at the third month. (*n* = 6 mice per group. Data are shown as mean ± SD.) (C) The line plots display the contribution of donor cells to PB overall (CD45.2^+^), B cell (B220^+^), T cell (CD3^+^), and myeloid (Mac-1^+^) every month after 30HSCs transplantation. (*n* = 6 mice per group. Data are shown as mean ± SD.) (D) Scheme of transplantation strategy for overexpression GSDMD in LSK cells. Freshly isolated LSK cells from WT mice were infected by lentivirus overexpression GSDMD, and 72 h later, 2 × 10^4^ GFP^+^ cells were purified and injected into lethally irradiated recipients together with 2 × 10^5^ competitor cells. (E) Representative western blot displays the expression of GSDMD in HEK293T cells’ lysates after vector-lentivirus or *Gsdmd*-OE-lentivirus infection. (F) The line plots display the contribution of donor cells to PB overall (CD45.2^+^), B cell (B220^+^), T cell (CD3^+^), and myeloid (Mac-1^+^) every month after transplantation. (*n* = 8 mice per group. Data are shown as mean ± SD.) (G) The lineage distribution of indicated donor-derived PB cells at the third month. (*n* = 8 mice per group. Data are shown as mean ± SD.) (H) Graphical summary.

Since targeted dysfunction of Gsdmd has no significant influence on the hematopoietic system and long-term hematopoietic reconstitution capacity of HSCs, we wondered whether enforced GSDMD affect the function of HSCs. To test this hypothesis, we cloned the cDNA of mouse Gsdmd into a lentivirus vector and it exhibits efficient overexpression (Fig. 2E). Freshly isolated LSK cells from WT mice were infected by GSDMD-carrying lentivirus, and 72 h later, 2 × 10^4^ GFP^+^ cells were purified and injected into lethally irradiated recipients together with 2 × 10^5^ competitor cells. Chimera in PB was checked every month until the third month (Fig. 2D). The lineage tracing show that the reconstitution capacity of HSCs and the lineage distribution of B cells and myeloid cells had no significant difference between GSDMD-overexpression and control groups (Fig. 2F), but the lineage distribution of T cells significantly is increased in GSDMD-overexpression group (Fig. 2G). Taken together, these results suggest that GSDMD overexpression does not affect the reconstitution capacity of HSC.

Taken together, we observed that targeted dysfunction of GSDMD did not affect the homeostasis of the blood system and the ability of HSCs to reconstitute the blood system (Fig. 2H). While, we observed that the number of CMP in *Gsdmd*^*−/−*^ mice is significantly higher than WT mice (Fig. 1F). Moreover, the fold change of myeloid cells of *Gsdmd*^*−/−*^ mice in response to LSP challenge is significantly higher than WT mice (Fig. 1M). Additionally, we observed that *Gsdmd*^*−/−*^ and overexpressed GSDMD exhibit unbalanced differentiation potential to T/B/Myeloid cells (Fig. 2B and 2G). Based on this data, we assume that GSDMD may play a function role in lineage differentiation at the steady state and in response to replication stress. Moreover, we observed that GSDME plays a functional role in HSCs by balancing pyroptosis and apoptosis [2]. It is possible that GSDME compensates the functional role of GSDMD in *Gsdmd*^*−/−*^. Therefore, double knockout mice (GSDMD/GSDME) are needed to elucidate this concern.

## Materials and methods

### Mice

*Gsdmd*^*−/−*^ mice were provided by Dr Feng Shao from National Institute of Biological Sciences. In all experiments (unless otherwise specified), mice were homozygous for *Gsdmd* allele. *Gsdmd*^*−/−*^ (C57BL/6, CD45.2) mice, C57BL/6-SJL (CD45.1) (Stock No: 002014), and C57BL/6 WT (CD45.2) (Stock No: 000664) mice were from the Jackson Laboratory. CD45.1/2 mice were heterozygotes from CD45.1 and CD45.2 mice. All mice were kept in specific-pathogen-free (SPF), AAALAC-accredited animal care facilities at the Laboratory Animal Research Center, Tsinghua University and all procedures were approved by Institutional Animal Care and Use Committee of Tsinghua University.

### Complete blood cell and BM cell count

PB was collected with an EDTA-containing tube from the tail and performed by the automatic hematology analyzer (BC-5000, Mindary). Single-cell suspension samples from BM were analyzed by Vi-CELL XR cell viability analyzer (Beckman Coulter).

### Flow cytometric analysis and cell sorting

All cells freshly isolated from mice were suspended in HBSS^+^ buffer supplemented with 2% fetal bovine serum, 1% penicillin/streptomycin, and 1% HEPES, and then stained with the indicated fluorochrome-labeled antibodies. Nonlysed BM cells were used for analysis of HSC and progenitor cells (antibodies including Lin-biotin cocktails, Streptavidin-APC/Cy7, Sca-1-PE/Cy7, c-Kit-APC, CD34-AF700, CD150-PE, CD135-PE-CF594, CD16/32-FITC, and CD127-BV421). Mature lineage cells from PB were pretreated by ACK buffer (KHCO_3_ 10 mM, NH_4_Cl 150 mM, Na_2_EDTA 0.1 mM, and adjust the pH 7.2–7.4) and stained with indicated antibodies (antibodies containing CD11b-Percpcy5.5, B220-PB, CD3-APC, CD45.1-FITC, and CD45.2-PE) before being subjected to flow cytometer for analysis. 10 ng/mL DAPI was used to indicate dead cells.

Flow cytometric analysis was performed with a BD LSRFortessa SORP flow cytometer (BD Biosciences) and data were analyzed using FlowJo™ Software (Becton, Dickinson and Company). BD Influx (BD Biosciences) was applied for cell sorting and the desired fractions were sorted into the indicated buffer.

### Competitive HSC transplantation

For the primary competitive HSC transplantation assay, 30 freshly isolated HSCs from WT or *Gsdmd*^−/−^ mice (CD45.2) were transplanted into lethally irradiated (10 Gy) recipients (CD45.1/2) together with 2.5 × 10^5^ competitor cells (CD45.1). 2 × 10^4^ GFP^+^ LSK cells (CD45.2) were transplanted into lethally irradiated (10 Gy) recipients (CD45.2) together with 2 × 10^5^ competitor cells (CD45.1). Donor-derived chimerism (including B cells, T cells, and myeloid) in PB of recipients were analyzed monthly interval.

### Lentivirus production and concentration

Lentivirus was packaged and concentrated as previously described [2]. Briefly, the Plasmid and helper plasmids were transfected to 293T cells using Polyethylenimine (Polysciences, 23966). Supernatants were collected at 48- and 72-h post-transfection and then concentrated (250×) by ultracentrifuge (Beckman, OPTIMA XE-90).

### Statistical analysis

Two-tailed unpaired Student’s *t* test was applied for analyzing experiment data after testing for normal distribution. All histogram and line graph were plotted by GraphPad Prism 7 software, and *P* < 0.05 was regarded as significant for all tests. All data are depicted as mean ± SD.
